# Comparing the inhalation of methacholine chloride and methacholine for methacholine challenge testing

**DOI:** 10.1186/s40001-026-03982-0

**Published:** 2026-02-04

**Authors:** Meiping Chen, Bin Shen, Peiling Feng, Yanping Wu, Gang Huang

**Affiliations:** Key Laboratory of Respiratory Disease of Zhejiang Province, Department of Respiratory and Critical Care Medicine, Second Affiliated Hospital of Zhejiang University School of Medicine, Hangzhou, 310009 Zhejiang China

**Keywords:** Asthma, Bronchial challenges testing, Bronchial hyperresponsiveness, Methacholine

## Abstract

**Background:**

Bronchial challenge testing with methacholine was applied to evaluate and quantify bronchial hyperresponsiveness (BHR) in asthma patients. We aimed to compare the clinical effectiveness between methacholine chloride and methacholine for methacholine challenge testing (MCT) in clinical practice, and investigate the adverse events associated with methacholine chloride.

**Methods:**

Patients who received methacholine and inhaled methacholine chloride for MCT were included in this retrospective study. All participants completed pulmonary function tests and MCT between January 2022 and February 2024. The provocative dose of methacholine that results in a 20% fall in FEV_1_ (PD20-FEV_1_) was used as quantitative measure of bronchial hyperresponsiveness. The primary outcome of the study was the proportion of positive MCT results and the degree of BHR, while the secondary outcome was the incidence rate of respiratory adverse events during methacholine chloride administration.

**Results:**

A total of 17,352 participants were included. In patients with bronchial symptoms, those administrated methacholine chloride for MCT demonstrated a significantly higher percentage of positive results compared with those administrated conventional methacholine (36.7% *vs.* 30.8%, *P* < 0.001). Moreover, PD20-FEV_1_ values were significantly lower in methacholine chloride group than methacholine group (*P* < 0.001). Chest tightness was the most prevalent adverse symptom affecting 30.8% of patients who received methacholine chloride, followed by cough at 27.2%. No serious adverse events were reported in these patients.

**Conclusions:**

The data indicated that methacholine chloride yielded a higher positive test rate than methacholine in MCT. Given that only mild adverse symptoms of chest tightness and cough were observed, with no serious adverse events reported, it represents a safe and effective alternative for clinical BHR assessment.

## Introduction

Asthma, which emerged as the most prevalent chronic respiratory disease globally in 2015, affecting over 300 million individuals, and stands as a leading cause of mortality and disability worldwide [1, 2]. This condition significantly compromises the quality of life and imposes a substantial financial burden. Bronchial challenge testing with methacholine has played an integral role in the evaluation and management of asthma [3]. As a direct stimulant, inhaled methacholine mimics the effect of neurotransmitter acetylcholine by acting on receptors in the airway smooth muscle, leading to airway contraction and narrowing [4].

The utilization of methacholine for bronchoprovocation originated and evolved in the mid-1940s [5]. However, conventional methacholine preparations are associated with concerns related to product impurities and variability. In contrast, Provocholine, a Food and Drug Administration (FDA) approved formulation of methacholine chloride in 1986, addressed issues such as deliquescence and ensured more consistent drug concentration, leading to its widespread adoption in many healthcare facilities across the United States [6]. Although studies reported good agreement between methacholine from industrial sources and Provocholine in terms of bronchoprovocation outcomes [7, 8], obtaining Provocholine in China remains a significant challenge. As a result, conventional methacholine has continued to be used for asthma diagnosis in both clinical and research settings.

The methacholine chloride formulation, which became available for methacholine challenge testing (MCT), was not approved in China until 2022 [9]. Currently, clinical data on this new formulation remain limited. Therefore, this study aims to compare the diagnostic efficacy of the novel methacholine chloride formulation versus conventional methacholine in asthma, and to evaluate the adverse events associated with the new formulation.

## Materials and methods

### Study design and participants

This retrospective study was conducted at the Second Affiliated Hospital of Zhejiang University School of Medicine, China. In this study, asthma was identified using International Classification of Diseases (ICD) codes [10]. Patients who received methacholine (methacholine, Sigma Chemical, St Louis, MO, USA) and used with novel inhaled methacholine chloride (methacholine chloride powder solution for solution, for inhalation, Tiangqingsuxin, Chai Tai Tianqing Pharmaceutical Group, Jiangsu, China) for MCT were included. Data from all participants aged ≥ 14 years who completed pulmonary function tests and MCT between January 2022 and February 2024 were retrieved from the clinical data system. Exclusions were: pregnancy, breastfeeding, age > 80 years, a fatal asthma attack history, forced expiratory volume in the first second (FEV_1_) < 60% or FEV_1_ < 1.0L, myocardial infarction or stroke in the past 3 months, uncontrolled hypertension (systolic > 200 mmHg, diastolic > 100 mmHg), recent eye surgery or raised intracranial pressure, asthma at acute stage, severe urticaria, inability to complete the testing, or incomplete clinical data.

Demographic data, pulmonary function (forced vital capacity [FVC], FEV_1_, FEV_1_/FVC%, MEF 50, MMEF 25, maximal mid-expiratory flow [MMEF 75/25]), the provocative dose of methacholine that results in a 20% fall in FEV_1_ (PD20-FEV_1_), and medical history were obtained.

This study was conducted in accordance with the Declaration of Helsinki, and the study protocol was approved by the Ethics Board of the Second Affiliated Hospital of Zhejiang University School of Medicine (2023–0875). All subjects provided written informed consent before conducting methacholine challenge testing.

### Spirometry and methacholine challenge testing (MCT)

Prior to conducting MCT, participants were required to undergo baseline spirometry assessment. All procedures were conducted in accordance with the standards set by the American Thoracic Society (ATS) [11]. All measurements were undertaken in triplicate, with a maximum deviation of less than 150 ml between the two best times in both FEV_1_ and FVC, and the highest value was selected as the reference for spirometry.

Both methacholine chloride and methacholine were dry crystalline powder. The solutions of doubling and quadrupling concentration were prepared by adding sterile saline diluent and following the manufacturer’s instructions. The MCT method was performed according to European Respiratory Society (ERS) [4] and Chinese Thoracic Society technical standard [12]. MCT with nebulized methacholine or methacholine chloride was performed by quantitative nebulization method, starting at low concentrations and gradually increasing to high concentrations in 2‐concentration, 6‐step method [12]. The FEV_1_ values and the cumulative dose at 30 s and 90 s after inhalation of methacholine or methacholine chloride were recorded. The participants who experienced a 20% decrease in FEV_1_ following MCT compared to their baseline measurements were classified as having a positive MCT test[12].

### Outcomes

The primary study outcome was the efficacy, assessed as the MCT response and the degree of bronchial hyperresponsiveness (BHR). The presence of a positive MCT result was defined as a PD20-FEV_1_ value less than 2.5 mg. The severity of BHR was classified as follows: severe BHR, PD20-FEV_1_ less than 0.035 mg, moderate BHR, PD20-FEV_1_ ranging from 0.035 to 0.293 mg, mild BHR, PD20-FEV_1_ ranging from 0.294 to 1.075 mg, and slight BHR, PD20-FEV_1_ ranging from 1.076 to 2.500 mg. The second outcome measured was the incidence rate of respiratory adverse events (including chest tightness, cough, wheeze, and pharyngeal irritation) assessed using MCT.

### Statistical analysis

For statistical analysis, IBM SPSS Statistics version 26.0. (SPSS Inc., Chicago, IL, USA), and GraphPad Prism 8 (GraphPad Software, San Diego, CA, USA) were used. Quantitative variables were presented as mean (standard deviation) or median (interquartile interval), and compared using Mann–Whitney U tests or two independent sample t-tests based on data distribution determined by Kolmogorov–Smirnov test. Categorical variables were expressed as percentages and analyzed using Chi-square tests for group comparisons. Statistical differences were considered at *P* < 0.05.

## Results

### Baseline characteristics

**Fig. 1:**
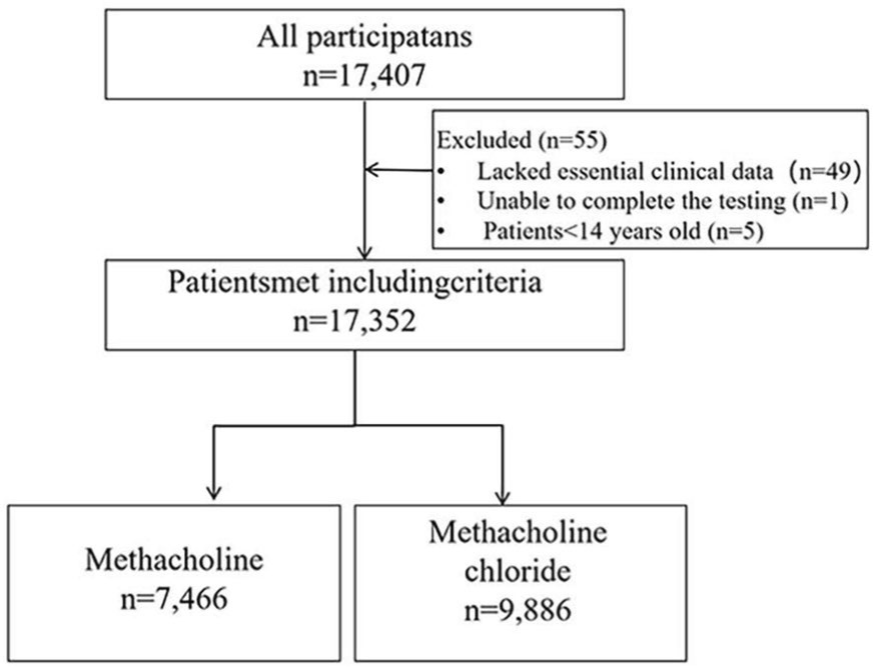
Flowchart of cohort selection

A total of 17,407 participants in this study completed spirometry and methacholine challenge testing. Out of these individuals, 55 were deemed ineligible for inclusion in this study due to various reasons: 49 lacked essential clinical data, one was unable to complete the required testing, and five were below the age threshold of 14 years. Consequently, a total of 17,352 participants were included for analysis. Specifically, comprehensive data were obtained from the methacholine group comprising 7466 patients while the remaining 9,886 belonged to the methacholine chloride group (Fig. 1). Most of the patients were female (63.4% *vs.* 63.0%) for these two groups. The mean age of methacholine group and methacholine chloride group was 41.6 and 41.5 years, respectively. The percentage of normal baseline spirometry was 82.2% for methacholine group and 81.4% for methacholine chloride group. No significant differences in gender, age, body mass index (BMI), or baseline spirometry were observed between the groups receiving methacholine and methacholine chloride (Table 1). A prior physician diagnosed history of asthma was documented in 17.8% of patients in the methacholine group, compared with 16.6% in the methacholine chloride group. In both cohorts, cough was found to be the most prevalent chief complaint, accounting for 59.1% of patients in the methacholine group and 56.3% in the methacholine chloride group.

**Table 1:** Demographics and clinical characteristics of all participants

	Methacholine *n* = 7466	Methacholine chloride group *n* = 9886	*P-value*
Gender, n (%)			0.593
Male	2730 (36.6)	3654 (37.0)	
Female	4736 (63.4)	6232 (63.0)	
Age, Mean (SD), y	41.6 (14.7)	41.5 (15.1)	0.542
BMI, Kg/m2, n (%)			0.279
< 18.5	628 (8.4)	780 (7.9)	
18.5–23.9	4170 (55.9)	5448 (55.1)	
24.0–27.9	2074 (27.8)	2830 (28.6)	
≥ 28	594 (8.0)	828 (8.4)	
Asthma history, n (%)	1330 (17.8)	1639 (16.6)	0.032
Chief complaint, n (%)			
Cough	4414 (59.1)	5561 (56.3)	< 0.001
Chest tightness	1180 (15.8)	1339 (13.5)	< 0.001
Allergic rhinitis	462 (6.2)	475 (4.8)	< 0.001
Abnormal baseline spiromety, n (%)			0.199
No	6134 (82.2)	8047 (81.4)	
Yes	1332 (17.8)	1839 (18.6)	
MCT results, n (%)			< 0.001
Positive	2301 (30.8)	3627 (36.7)	
Negative	5165 (69.2)	6259 (63.3)	

*BMI* body mass index, *MCT* methacholine challenge test. *P* < 0.05 was considered as statistically significant

### Differences of methacholine and methacholine chloride for MCT

**Fig. 2:**
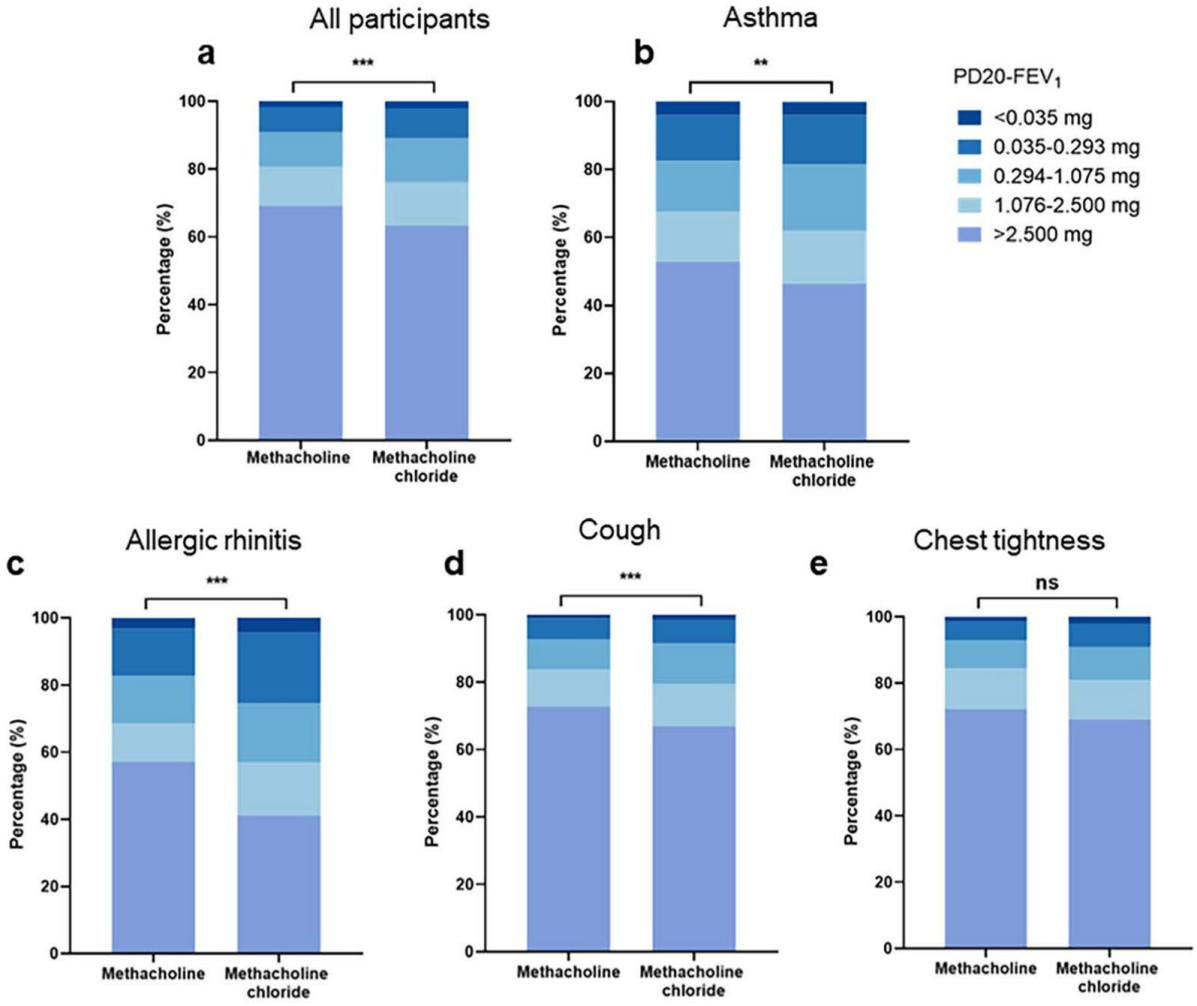
Severity of AHR in patients receiving methacholine chloride and Methacholine **a** All participants; **b** Patients with a history suggestive of asthma; **c** Patients with allergic rhinitis; **d** Patients with cough symptom; **e** Patients with chest tightness symptom; *P < 0.05, **P < 0.01, ***P < 0.001

Patients administrated with methacholine chloride (36.7%) exhibited a higher percentage of positive MCT results (indicating PD20-FEV_1_ ≤ 2.5 mg) compared to those administrated with methacholine (30.8%, *P* < 0.001, Table 1). Moreover, PD20-FEV_1_ value was significant lower in methacholine chloride group than methacholine group (*P* < 0.001, Fig. 2a), indicating that individuals administered with methacholine chloride demonstrated a higher degree of BHR when compared to members with methacholine.

Comparisons between groups based on patients with a history suggestive of asthma were presented in Table 2. The baseline characteristics, including the proportion of female patients, age, BMI distribution, and baseline spirometry results, exhibited no significant differences between individuals receiving methacholine chloride (*n* = 1639) and those administered with methacholine (*n* = 1330). Notably, the proportion of positive MCT results was significant higher in patients who underwent methacholine chloride (53.6%) than those who received methacholine (47.1%,* P* < 0.001, Table 2). Furthermore, a significant decrease in PD20-FEV_1_ value was observed in individuals administrated with methacholine chloride (*P* = 0.002, Fig. 2b). A higher proportion of positive MCT results was observed in patients with allergic rhinitis (AR) who received methacholine chloride (58.9%) compared to those who received methacholine (42.9%, *P* < 0.001, Supplementary Table 1). In addition, a significantly decreased in PD20-FEV_1_ value was also noted in methacholine chloride group than methacholine group (*P* < 0.001, Fig. 2c).

**Table 2:** Demographics and clinical characteristics of patients with a history of asthma

	Methacholine *n* = 1330	Methacholine chloride group *n* = 1639	*P-value*
Gender, n (%)			0.287
Male	445 (33.5)	579 (35.3)	
Female	885 (66.5)	1060 (64.70)	
Age, Mean (SD), y	39.3 (14.6)	38.5 (15.2)	0.070
BMI, Kg/m2, n (%)		s	0.265
< 18.5	100 (7.5)	152 (9.3)	
18.5–23.9	781 (58.7)	948 (57.8)	
24.0–27.9	341 (25.6)	424 (25.9)	
≥ 28	108 (8.1)	115 (7.0)	
Abnormal baseline spiromety, n (%)			0.741
No	978 (73.5)	1214 (74.1)	
Yes	352 (26.5)	425 (25.9)	
MCT results, n (%)			< 0.001
Positive	627 (47.1)	878 (53.6)	
Negative	703 (52.9)	761 (46.4)	

*P* < 0.05 was considered as statistically significant

Both cough variant asthma (CVA) and chest tightness variant asthma (CTVA) are the main subtypes of atypical asthma. Therefore, an analysis was conducted in patients presenting with chief complaints of chest tightness and cough. The proportion of positive MCT results was higher in the methacholine chloride group (33.1%) compared to the methacholine group (27.2%, *P* < 0.001, Supplementary Table 2). Furthermore, we found that individuals in the methacholine chloride group exhibited lower PD20-FEV_1_ value than those in the methacholine group in patients with cough symptoms (*P* < 0.001, Fig. 2d).

However, there is an upward trend observed in the methacholine chloride group, despite no significant difference being found in the proportion of positive MCT results between both groups of patients presenting with chest tightness (methacholine chloride *vs.* methacholine, 31.1% *vs.* 27.9%, *P* = 0.074, Supplementary Table 3).

### Adverse respiratory events of methacholine chloride for MCT

**Fig. 3:**
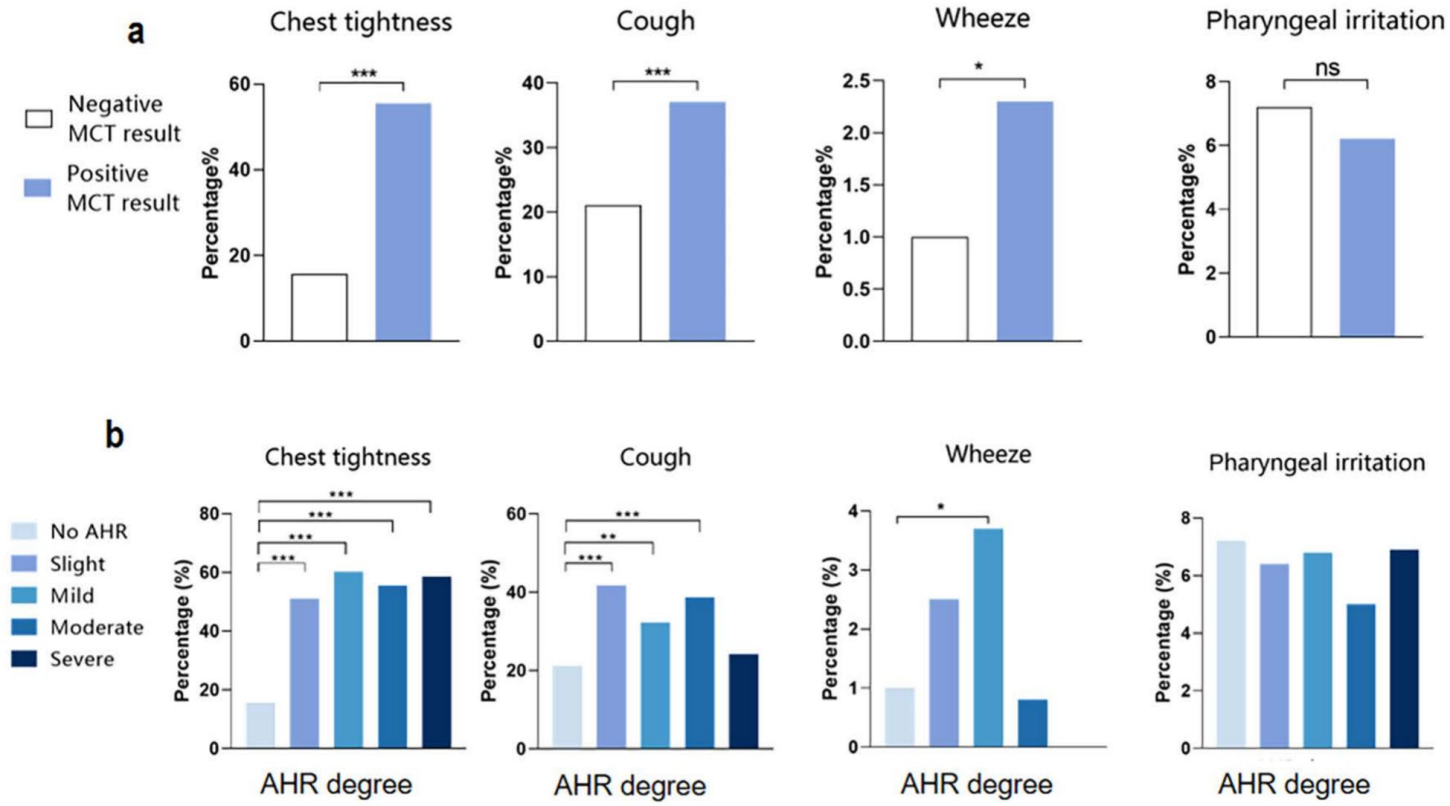
Incidence of respiratory adverse events in patients receiving methacholine chloride. **a** Proportion of adverse events in both positive and negative MCT result groups; **b** Distribution characteristics of adverse events in different degrees of BHR. *P < 0.05, **P < 0.01, ***P < 0.001

To further investigate the occurrence of adverse events in patients receiving methacholine chloride, we recorded respiratory symptoms during MCT procedures (*n* = 1460, Supplementary Fig. 1). During MCT administration, the reported adverse respiratory events, such as chest tightness, cough, wheezing, and pharyngeal irritation, accounted for 58.0% of patients. Chest tightness was the most prevalent adverse symptom at 30.8%, followed by cough at 27.2% (Supplementary Fig. 1). There were no serious adverse events reported, such as mortality or transfer to the emergency department. Patients with positive MCT results exhibited a significantly higher frequency of chest tightness (55.4%) compared to those with negative results (15.7%, *P* < 0.001, Fig. 3a). Moreover, individuals receiving methacholine chloride who tested positive on MCT displayed elevated incidences of both cough and wheezing (cough, 37.0% *vs.* 21.1%, *P* < 0.001; wheeze, 2.3% *vs.*1.0%, *P* < 0.05, Fig. 3a). Additionally, patients exhibiting various degrees of BHR demonstrated significantly higher incidences of chest tightness and cough compared to those without any BHR (Fig. 3b).

## Discussion

Our analysis showed that the methacholine chloride group demonstrated a higher proportion of positive MCT results in comparison to the methacholine group. Furthermore, patients with asthma, AR, and cough who underwent the methacholine chloride test demonstrated both a greater occurrence of positive MCT results and a higher degree of BHR when compared to those who underwent methacholine test. Moreover, over 50% of the patients reported experiencing adverse respiratory events following MCT administration, with chest tightness and cough being the most frequently reported symptoms.

Methacholine, as a direct stimuli muscarinic agonist, stimulates airway smooth muscle via the muscarinic (M3) receptor, leading to airway narrowing, increased air flow resistance, and resulting reduction in FEV_1_ in individuals with asthma [13, 14]. Despite there being no gold standard test to detect asthma, the evaluation and quantitation of BHR is recommended by American Thoracic Society as one of the best methods for diagnosing asthma [15]. Although methacholine is used to assess the prevalence of BHR in various studies, its significance in asthma diagnosis has attracted enormous attention. Romero et al. [16] evaluated individuals presenting with respiratory symptoms, normal baseline spirometry, and negative bronchodilator test. They observed that 30.6% of these individuals tested positive for methacholine chloride, with a MCT sensitivity of 59.3% and specificity of 98.1% [16]. Boulet et al*.* reported that 36.0% of subjects with normal pre-bronchodilator spirometry and chronic respiratory symptoms exhibited airway hyperresponsiveness to methacholine chloride (defined as concentration causing a 20% fall in FEV_1_ [PC20] < 16 mg/ml) [17]. Louis et al*.*'s study showed that of 194 asthma patients with baseline FEV_1_ > 70% of predicted, 71% of patients displayed positive MCT (PC20 < 16 mg/ml) to methacholine chloride [18]. Here, we found that 36.7% positive MCT result in methacholine chloride group among patients presenting with respiratory symptoms. The findings are in line with the research conducted by Romero et al*.* However, we offer an analysis of the distribution characteristics of BHR, and display the results of MCT in patients presenting with diverse respiratory symptoms.

It is noteworthy that patients underwent methacholine chloride administration, which resulted in relatively decreased PD20-FEV_1_ compared to methacholine. We observed that methacholine chloride may outperform methacholine for MCT without selecting patients based on distinct subtype asthma. However, there is limited data comparing Provocholine and methacholine. The comparison between Provocholine and methacholine was first reported by Sherman in 1994 [8]. A double blind, cross-over clinical study was conducted, which showed no significant difference in the results after challenges with methacholine and Provocholine. No significant difference was observed in structural composition by proton beam nuclear magnetic resonance [8]. Moreover, Ferrari et al. found excellent agreement in the MCT between Provocholine and methacholine, and no significant difference structurally [7]. Therefore, we had to opt for methacholine for MCT in clinical practice due to the unavailability of Provocholine in China. To the best of our knowledge, this study sample size comparing methacholine chloride and methacholine is the largest in clinical practice in China. Our results diverge from studies conducted over 20 years ago, possibly due to their limited sample size and the majority of participants lacking potential asthma symptoms [7]. Moreover, nonuniform types of nebulizer systems and the use of dose–response slope as the main results for MCT may have contributed to these discrepancies [19, 20].

Respiratory symptoms are a recognized feature of patients undergoing MCT [4]. A study with a small sample size reported about 6.7% of adults with asthma experience adverse respiratory events and decreased oxygen during MCT. Additionally, adverse events such as cough (40%) and dyspnea (10%) were observed in asthmatic children [21]. Covar's investigation on children found that 7% of those with positive and incomplete challenge experienced severe adverse events, with chest tightness, cough, and shortness of breath being the most common symptoms [22]. In the present study, we found that approximately one third of participants experienced chest tightness and coughing during methacholine chloride administration, but without any serious adverse effects.

Methacholine chloride, as the first agent approved for bronchial provocation tests in China, has been well standardized in clinical practice domestically [12]. Mechanistically, as a direct cholinergic receptor agonist, inhaled methacholine chloride acts specifically on airway smooth muscle cells, rendering it a sensitive tool for detecting BHR. It is thus crucial for comprehensively assessing the airway response status of the target population, including individuals with atypical symptoms. A key practical advantage of methacholine chloride is that it overcomes the deliquescence issue associated with conventional methacholine, thereby ensuring consistent drug concentrations [23]. Furthermore, the use of methacholine chloride allowed this study to be directly compared with the vast majority of previous studies conducted in accordance with the guidelines of the American Thoracic Society/European Respiratory Society (ATS/ERS) [4]. Moreover, no serious adverse events were observed during MCT. Therefore, we proposed that this newly approved methacholine chloride in China is a safe and relatively conventional method for measuring BHR.

One limitation of this study is the absence of repeated MCT with methacholine or methacholine chloride. However, this is a retrospective study with thousands of MCT. We also have insufficient data on adverse events related to the methacholine challenge. Additionally, there was no follow-up on these patients to determine if they were eventually diagnosed with asthma by clinicians after MCT. Moreover, since methacholine and methacholine chloride were not directly compared, the superiority or inferiority can only be determined indirectly. The medication for asthma can affect bronchial hyperresponsiveness measurement, but because this study is a database analysis, the detailed treatment status is unknown. Therefore, further exploration is needed to determine the actual value of methacholine chloride in diagnosing asthma.

## Conclusion

In this study, we observed that patients administered with methacholine chloride for MCT exhibited a higher incidence of positive MCT results and a higher degree of BHR compared to those administered with methacholine in patients with bronchial symptoms. Moreover, approximately one-third of participants experienced chest tightness and coughing during methacholine chloride administration, but without any serious adverse effects.

## Data Availability

The data that support the findings of this study are available from the corresponding author upon reasonable request.

## Abbreviations

BHR: Bronchial hyperresponsiveness
BDR: Bronchodilator responsiveness
CVA: Cough variant asthma
CTVA: Chest tightness variant asthma
FDA: Food and drug administration
FVC: Forced vital capacity
FEV_1_: Forced expiratory volume in the first second
ICD: International classification of diseases
MCT: Methacholine challenge testing
MMEF 75/25: Maximal mid-expiratory flow
PD20-FEV_1_: Provocative dose of methacholine that results in a 20% fall in FEV1
PC20: Concentration causing a 20% fall in FEV_1_
